# Impact of hospital safety culture and workplace sexual harassment on turnover intention of nursing professionals in Upper East Region, Ghana

**DOI:** 10.1186/s12912-026-05204-8

**Published:** 2026-08-07

**Authors:** Charles Lwanga M. Tabase, Lisa C. Barry, Edward W. Ansah

**Affiliations:** 1 Nursing and Midwifery Training College-Zuarungu, Bolgatanga, Upper East Region Ghana; 2 Center on Aging, University of Connecticut Health Center, Farmington, CT USA; 3 Department of Health, Physical Education and Recreation, University of Cape Coast, Cape Coast, Ghana

**Keywords:** Safety culture, Sexual harassment, Turnover intention, Nursing professionals, Healthcare facilities

## Abstract

**Background:**

The global nursing professionals’ shortfall is alarming, reaching 4.81 million by 2030, with Africa and South-East Asia disproportionately affected. While improved organizational safety culture promotes worker retention, unsafe work environments, including sexual harassment, contribute to high turnover rates and associated decrease in healthcare service quality. However, the literature is limited on how hospital safety culture and sexual harassment of nursing professionals affect their turnover intention rates in rural settings.

**Methods:**

We conducted a cross-sectional study to evaluate the relationships among safety culture, sexual harassment, and turnover intention among nursing professionals (*N* = 812) in the Upper East Region of Ghana. Participants completed an online survey that included the 25-item Airline Safety Culture Index, the 17-item Sexual Experience Questionnaire, and the 6-item Turnover Intention Scale. Descriptive statistics summarized participants’ characteristics and the variables under study. Chi-square test assessed bivariate associations, and multivariable binary logistic regression estimated the association between safety culture, sexual harassment and turnover intention, adjusting for years of working experience and sex. Sex-stratified analysis was also performed.

**Results:**

Results indicate that 7.5%, 63.1% and 29.4% of the workers rated their hospitals as having a low, moderate, and high safety culture, respectively. Overall prevalence of workplace sexual harassment was 30.7%; gender harassment 27.3%, unwanted sexual harassment 50.9%, and sexual coercion 49.8%, with 48.2% of these workers intending to leave their current positions in the very near future. Perception of safety culture within healthcare facilities, years of work experience, sexual harassment, and sex are associated with turnover intention among nursing professionals. Among females, sexual harassment predicted turnover intention, whereas among males, years of experience and safety culture were significant predictors.

**Conclusion:**

Immediate action is needed to enhance the organizational safety culture of these healthcare facilities, addressing workplace sexual harassment, reducing sex stereotypes, and preventing further depletion of this critical healthcare workforce. Without immediate action, this could severely affect patient outcomes and healthcare quality in Ghana, thereby compromising progress toward Sustainable Development Goal 3.

**Clinical trial number:**

Not applicable

## Introduction

Nursing professionals are the majority of the healthcare workforce globally, having crucial roles in patient care, including emergency response and public health services. However, recent estimates indicate a global shortage of about 4.81 million nursing professionals (i.e., 4.5 million nurses and 0.31 million midwives) by 2030 [1]. The nursing shortage may be especially detrimental in low-income countries (LICs), such as Africa and the Mediterranean regions, where healthcare resources are often scarce, and large rural communities are prevalent. This is a substantial public health problem considering that LICs are expected to account for approximately 70% of the global nursing shortage by 2030 [2]. Consequently, the nursing shortage may compromise Universal Health Coverage (UHC) and the United Nations’ Sustainable Development Goals (SDGs), particularly SDG 3, which aims to ensure healthy lives and promote well-being for all ages [2–4]. The shortage may be particularly problematic in an underserved region like Ghana’s Upper East Region. This region is among the poorest in the country, with nearly 90% of its population living in rural, hard-to-reach areas and lacking basic healthcare equipment and professional services [5]. It is important to improve understanding of the factors that may impact the nursing shortage in these areas so that they can be addressed.

Turnover is a noted primary reason for the global nursing shortage [6–9]. Recent global reviews and meta-analyses report turnover intention rates among nursing professionals ranging from 18% to 38.4% [10–12]. In Ghana, turnover rates among nursing professionals are especially high, ranging from 48.9% to 87.2% across southern regions and teaching hospitals [13–15]. Turnover has significant negative consequences, including increased stress for the remaining hospital staff, diminished quality of patient care, and increased costs to recruit and train new workers [16, 17]. In rural settings, such as the Upper East Region of Ghana, where recruiting and retaining healthcare staff can be challenging, the negative implications of turnover may be even more severe, with significant consequences for vaccination coverage, the treatment of communicable and non-communicable diseases, and maternal and infant mortality in the region. Thus, reducing turnover is important.

One way to reduce turnover may be to improve the organization’s safety culture. Organizational safety culture refers to shared, agreed-upon practices within an organization, such as how things are done and the mentality that guides thinking and controls risks associated with activities [18]. There are three types of safety culture. The first, and least preferred, is a pathological safety culture, where the organization prioritizes productivity over worker and patient safety and well-being. The second type, bureaucratic safety culture, refers to unquestioning adherence to safety rules and procedures, with an inadequate understanding of their implications due to a lesser emphasis on open communication, error reporting, and adaptive problem resolution. The third type, generative safety culture, is ideal and considered a positive safety culture; where organizations proactively seek to enhance safety through shared responsibilities and continuous learning by both workers and management [19, 20].

One factor that may impact workers’ perceptions of safety culture is workplace sexual harassment. Workplace sexual harassment can be categorized as unwanted sexual harassment, i.e., stroking, touching, or any unwelcome request for sexual or romantic relationships; sexual coercion, i.e., threatening or frightening behavior towards any worker to urge cooperation/favour or as a form of maltreatment or a bribe for sexual advances; and gender harassment, i.e., physical or verbal behavior that depicts insulting, degrading, or sexist remarks about the opposite sex [21–23]. Prior studies reported that workplace sexual harassment among nursing professionals is common, especially in facilities with poor safety culture [21, 24]. Moreover, evidence suggests that workplace sexual harassment is associated with turnover intentions [21, 25, 26]. However, studies linking hospital or healthcare facility safety culture and sexual harassment with turnover and turnover intention are limited. For instance, evidence on whether turnover intention is associated with perceptions of safety culture and sexual harassment in underserved regions in Ghana is unknown. Therefore, the present study evaluates the relationships among safety culture, reports of sexual harassment, and turnover intention among nursing professionals in the Upper East Region of Ghana. This information may have important implications for developing and implementing targeted interventions to stabilize the healthcare workforce and ultimately enhance healthcare quality in these rural and low-income areas.

## Method and materials

### Study design and setting

This is a quantitative cross-sectional survey that examined the relationships among safety culture, sexual harassment, and turnover intention among nursing professionals working in healthcare facilities in the Upper East Region of Ghana. The design provides a ‘snapshot’ of the phenomenon under study within a specified period [27]. It allows researchers to collect and access primary data to estimate the prevalence of all variables, provide a critical baseline, and assess associations [28–31]. The design was used in similar studies in recent time [28]. An online survey approach was utilized to help eliminate the time and cost of sending the questionnaire to participants across the region, the time used in entering the data from all nurses, ease the administering of the instruments compared to the paper survey, promote data accuracy, and reduce the printing cost involved in the paper survey [32].

Those eligible for the study included nursing professionals (i.e., registered general nurses, registered midwives, registered mental health nurses, registered community nurses, nurse assistants clinical, nurse assistants preventive, and other nursing specialties) who worked in any public healthcare facility in Ghana’s Upper East Region under government payroll, particularly Ghana Health Service (GHS) and Christian Health Association of Ghana (CHAG) for at least one year. This region, located in the northern part of the country, comprises 15 districts and municipalities, with Bolgatanga as the capital city [33]. Healthcare in this region is delivered via a four-level system (community, sub-district, district, and regional). Apart from the regional hospital, which provides tertiary healthcare services, there are 6 GHS district hospitals, 3 CHAG, and 10 private hospitals that provide secondary-level healthcare in the region. There are 38 clinics, 67 health centers, about 1107 Community Health Planning and Services (CHPS) facilities, and 3 private maternity homes [34, 35]. In 2017, it had an inpatient attendance of 83,919 and a total bed capacity of 1,363 [35].

The region has approximately 3,660 nursing professionals. We distributed the online survey to all these nursing professionals between May 9, 2022 and August 19, 2022 via the WhatsApp platforms of the Union of Professional Nurses and Midwives, Ghana (UPNMG) and the Ghana Registered Nurses and Midwives Association (GRNMA). These common WhatsApp platforms are available for all members. However, only 812 (22.2%) responded, for which we could use their data for this study. Despite the reported 22.2% response rate among nursing professionals, the absolute sample of 812 is large enough and could represent nursing professionals in the region. This is because the majority of these workers are believed to have smartphones, which they could use to complete the survey within 3 months. To enhance response rates, researchers sent routine reminders to nursing professionals via their leaders using the WhatsApp form. The cover page of the survey specified that participants were to complete the questionnaire only once. In accordance with the Declaration of Helsinki, an informed consent form attached to the survey detailed the study’s purpose, estimated completion time, voluntary participation and the right to withdraw, confidentiality and anonymity assurances, and potential risks. Participants provided informed consent prior to completing the survey.

### Measures

#### Safety culture

Organizational safety culture was assessed using a modified version of the 25-item Airline Safety Culture Index [ASCI] [36]. It has a reliability coefficient alpha of 0.81 [37]. In studying mental health nurses in Ghana, Sarfo [38] adapted the same instrument where words such as ‘company’ and ‘airline’ were substituted with ‘institution/hospital/facility, ‘work area,’ and ‘employee’ with ‘ward/department’ and ‘nurse’ to fit the current study. Moreover, a recent psychometric analysis of the ASCI using data from nurses serving at psychiatric hospitals in Ghana also yielded a Cronbach’s alpha reliability coefficient of 0.85 [38]. The ASCI uses a 5-point Likert scale: 1 = Strongly Disagree, 2 = Disagree, 3 = No Idea, 4 = Agree, and 5 = Strongly Agree. A composite safety culture score was computed (ranging from 25 to 125), with higher scores indicating better safety culture [19]. Safety culture scores were categorized as pathological (25 to 58), bureaucratic (59 to 92), or generative (93 to 125) [36].

### Sexual harassment

The 17-item Sexual Experience Questionnaire [SEQ] [39], with a reliability coefficient alpha of 0.94 [40], was used to assess the level of sexual harassment. The SEQ is scored using a 5-point Likert scale, from “Never” [1] to “Once” [2], “Sometimes” [3], “Often” [4], to “Most of the time” [5]. Overall scores and subscale scores (unwanted sexual attention, sexual coercion, and gender harassment) increase as the frequency of sexual harassment increases. For the following established procedure [41–43], sex-specific average scores were calculated for the overall scale and for each of the three subscales. Participants scoring above the mean score were categorized as experiencing high sexual harassment [39].

### Turnover intention

Nursing professionals’ turnover intention was measured using the 6-item Turnover Intention Scale [TIS-6] [44]. The TIS-6 uses a 5-point Likert scale from Never [1] to Always [5], with reliability coefficients of 0.80 [44] and 0.88 [45]. All items were summed to a possible score of 1 to 30, and scores of 18 or higher indicated high intent to leave/high turnover intention [46, 47].

### Additional variables

We also collected information on participants’ demographics, including age, sex, years of experience, highest educational level, marital status, nursing professional category, shift, and work district.

### Approval to conduct the study

Institutional Review Board of the University of Cape Coast (ID- UCCIRB/CES/2021/167) and Navrongo Health Research Center (ID-NIIRCIRB468) granted ethical clearance for the study. We also obtained permission from all public healthcare facilities, including the Regional, Municipal/District Directorates of GHS and CHAG, and from all facility heads.

### Data analysis

Completed online surveys were downloaded from the online platform (Google Forms) into a password-protected Microsoft Excel sheet prior to being uploaded into the IBM SPSS software package (Version 26). Data cleaning was performed manually and electronically by screening for completeness in Microsoft Excel and in IBM SPSS. Data were complete, with no missing values, possibly due to the online survey approach, which included prompts to complete each section before moving to the next. Age in years was categorized into three (20–34, 35–49, and ≥ 50), years of working experience categorized into three (1–5, 6–10, and ≥ 11), category of staff into three (professional, auxiliary, and other), and districts or municipalities categorized into urban and rural.

Frequency and percentage analyses, means, and standard deviations were used to determine levels of hospital safety culture, sexual harassment, and turnover intention among the study participants. Chi-square tests were used to assess the bivariate association between independent variables, sexual harassment (categorized based on sex-specific mean scores), safety culture, and the dependent variable, turnover intention. We used binary logistic regression to determine the association between safety culture and sexual harassment and turnover intention, controlling for years of work experience and sex. Years of experience and sex were included in the model due to their theoretical and empirical relevance to both safety culture and sexual harassment and turnover intention. The covariates were limited to optimize the model parsimony while still controlling for important plausible confounds. We evaluated model fit using the Hosmer-Lemeshow goodness-of-fit test, the Omnibus test, and R^2^ statistics (Nagelkerke, Cox, and Snell). We repeated these analyses after stratifying by sex. All analyses were conducted using IBM SPSS Statistics version 26.0, with p-values < 0.05 and a 95% confidence interval used to report statistically significant results.

## Results

### Demographic characteristics of respondents

A total of 812 (22.2%) nursing professionals responded to the online survey. Table 1 presents the characteristics of the study participants. The sample ranged from age 20 to 56 years (M = 32.06; SD = 4.66) and was 50.7% male. Approximately 30% of respondents were no more than 25 years from the mandatory retirement age of 60 years, and professional nurses comprised the highest proportion of respondents. Close to half (45.3%) of the participants have 1–5 years of work experience, 56.2% worked both day and night shifts, and 46.6% held a diploma.

**Table 1:** Demographic Characteristics of Respondents

Characteristics	Variable(s)	Total *n* (%)
Sex	Male	412 (50.7)
	Female	400 (49.3)
Age	20 to 34	569 (70.1)
	35 to 49	241 (29.7)
	≥ 50	2 (0.2)
Years of work experience	1 to 5	368 (45.3)
	6 to 10	301 (37.1)
	≥ 11	143 (17.6)
Shift	Day only	348 (42.9)
	Night only	8 (1.0)
	Day and night	456 (56.2)
Category of nursing professionals	Professional^1^	555 (68)
	Auxiliary^2^	183 (22.5)
	Other^3^	77 (9.5)
Highest educational level	Bachelors	232 (28.6)
	Certificate	180 (22.2)
	Diploma	378 (46.6)
	Masters	22 (2.7)
Marital status	Single	258 (31.8)
	Married	540 (66.5)
	Divorce/Separated	9 (1.1)
	Widow/Widower	5 (0.6)
District/Municipality	Urban^4^	419 (51.6)
	Rural^5^	393 (48.4)

^1^Professional includes Registered General Nurses, Registered Midwives, Registered Mental Nurses, and Registered Community Nurses

^2^Auxiliary includes Nurse Assistant Clinical and Nurse Assistant Preventive

^3^Other includes Eye, Oral, Emergency, Pediatric, Critical Care, Ear Nose and Throat nurses and other specialist

^4^Urban includes the municipals (Bawku, Bolgatange, Bulisa North, and Kassena Nankana)

^5^Rural includes the districts (Bawku West, Binduri, Bolgatanga East, Bongo, Bulisa South, Garu, Kassena Nankana West, Nabdam, Pusiga, Talensi, and Tempane

### Level of safety culture, sexual harassment and turnover intention

Table 2 describes safety culture, sexual harassment and turnover intention reported by study participants. The results showed a mean turnover intention score of 18.17 (SD = 4.50) and 48.2% intended to leave their facilities in the very near future. The average safety culture score was 82.60 (SD = 15.93). Approximately 29% of respondents reported a Generative/High Safety Culture. The majority (63.1%) of workers considered their facilities to have a bureaucratic/medium safety culture, and less than 8% reported a pathological/low safety culture. The overall sample mean score of sexual harassment was 22.44 (SD = 8.34), and a total of 30.7% of the participants experienced a high level of sexual harassment at work. Regarding the subscales of sexual harassment, the majority of participants (50.9%) reported experiencing high unwanted sexual attention, nearly half (49.8%) reported high level of sexual coercion, and over one-quarter (27.3%) reported gender harassment. While average overall sexual harassment scores, as well as subscale scores, were higher among females than males, these differences were not statistically significant. All variables demonstrated high reliability coefficients, i.e., safety culture (Cronbach’s alpha = 0.925), sexual harassment (Cronbach’s alpha = 0.921), and turnover intention (Cronbach’s alpha = 0.678). At the subscale level of sexual harassment, Cronbach’s alpha was 0.787 for gender harassment, 0.873 for unwanted sexual attention, and 0.834 for sexual coercion.

**Table 2:** Descriptives of Turnover Intention, Safety Culture, and Sexual Harassment

Variable	*n* (%)	Overall Mean (SD^6^)	Male Mean (SD)	Female Mean (SD)
Turnover intention				
Low	421 (51.8)	18.17 (4.50)		
High	391 (48.2)			
Safety culture				
Pathological/Low	61 (7.5)	82.60 (15.93)		
Bureaucratic/Medium	512 (63.1)			
Generative/High	239 (29.4)			
Sexual harassment				
Low	563 (69.3)	22.44 (8.34)	21.79 (7.01)	23.11 (9.49)
High	249 (30.7)			
Gender harassment				
Low	590 (72)	7.15 (3.00)	7.09 (2.82)	7.21 (3.17)
High	222 (27.3)			
Unwanted sexual attention				
Low	399 (49.1)	23.32 (4.96)	23.06 (4.72)	23.59 (5.19)
High	413 (50.9)			
Sexual coercion				
Low	408 (50.2)	15.25 (3.97)	15.06 (3.90)	15.45 (4.04)
High	404 (49.8)			

^6^SD, Standard deviation

### Bivariate results

Table 3 presents the association between safety culture and turnover intention, stratified by sex. Overall, turnover intention increased as safety culture worsened (χ² = 20.31 [2], *p* < 0.001). The majority of participants reported a bureaucratic safety culture, whereas a few reported a pathological safety culture. Among male nursing professionals, a significantly higher proportion of those reporting pathological safety culture (71%) and bureaucratic safety culture (60.9%) had higher turnover intention compared with those reporting generative safety culture (38%) (χ² = 19.55 [2], *p* < 0.001). The trend is similar among females; however, it is not significant (χ² = 3.15 [2], *p* < 0.21).

Using sex-specific mean scores for the bivariate association between sexual harassment and turnover intention, overall sexual harassment was significantly associated with turnover intention (χ² = 8.02 [1], *p* = 0.005). Using sex-specific means, a total of 252 (31%) of the participants experienced high level of sexual harassment at work, with 32.5% of males and 29.5% of females experiencing high sexual harassment, respectively. Sexual harassment was associated with turnover intention among females, with those experiencing high sexual harassment more likely to report high turnover intention (χ² = 6.27 [1], *p* = 0.01). This association was not significant among the males (χ² = 1.90 [1], *p* = 0.17).

The average years of work experience for the overall sample was 6.56 (SD = 4.54), with the majority reporting 1–5 years of experience. Overall, greater years of working experience was associated with high turnover intention (χ² = 12.81 [2], *p* < 0.004). However, sex-specific analysis revealed that years of work experience was only associated with turnover intention among males (χ² = 10.91 [2], *p* = 0.002).

**Table 3:** Chi-square analysis of safety culture, sexual harassment and working experience with turnover intention

	Variable	Low TOI^7^ *n* (%)	High TOI *n* (%)	Total *n* (%)	*p*-value	χ²(df)
	**Level of Safety Culture (SC)**					
Male	Pathological/Low SC	9 (29.0)	22 (71.0)	31 (7.5)	< 0.001	19.55 (2)
	Bureaucratic/Medium SC	106 (39.1)	165 (60.9)	271 (65.8)		
	Generative/High SC	68 (61.8)	42 (38.2)	110 (26.7)		
	**Total**	**183 (44.4)**	**229 (55.6)**	**412**		
Female	Pathological/Low SC	15 (50.0)	15 (50.0)	30 (7.5)	0.208	3.15 (2)
	Bureaucratic/Medium SC	139 (57.7)	102 (42.3)	241 (60.2)		
	Generative/High SC	84 (65.1)	45 (34.9)	129 (32.3)		
	**Total**	**238 (59.5)**	**162 (40.5)**	**400**		
Overall Total	Pathological/Low SC	24 (39.3)	37 (60.7)	61 (7.5)	< 0.001	20.31 (2)
	Bureaucratic/Medium SC	245 (47.9)	267 (52.1)	512 (63.1)		
	Generative/High SC	152 (63.6)	87 (36.4)	239 (29.4)		
	**Total**	**421 (51.8)**	**391 (48.2)**	**812**		
	**Level of Sexual Harassment (SH)**					
Male	Low SH	130 (46.8)	148 (53.2)	278 (67.5)	0.17	1.90 (1)
	High SH	53 (39.6)	81 (60.4)	134 (32.5)		
	**Total**	**183 (44.4)**	**229 (55.6)**	**412**		
Female	Low SH	179 (63.5)	103 (36.5)	282 (70.5)	0.01	6.27 (1)
	High SH	59 (50.0)	59 (50.0)	118 (29.5)		
	**Total**	**238 (59.5)**	**162 (40.5)**	**400**		
Overall Total	Low SH	309 (55.2)	251 (44.8)	560 (69.0)	0.005	8.02 (1)
	High SH	112 (44.4)	140 (55.6)	252 (31.0)		
	**Total**	**421 (51.8)**	**391 (48.2)**	**812**		
	**Years of Work Experience (YoWE)**					
Male	1 ~ 5	83 (55.0)	68 (45.0)	151 (36.7)	0.004	10.91 (2)
	6 ~ 10	75 (39.1)	117 (60.9)	192 (46.6)		
	≥ 11	25 (36.2)	44 (63.8)	69 (16.7)		
	**Total**	**183 (44.4)**	**229 (55.6)**	**412**		
Female	1 ~ 5	133 (61.3)	84 (38.7)	217 (54.2)	0.415	1.76 (2)
	6 ~ 10	66 (60.6)	43 (39.4)	109 (27.3)		
	≥ 11	39 (52.7)	35 (47.3)	74 (18.5)		
	**Total**	**238 (59.5)**	**162 (40.5)**	**400**		
Overall Total	1 ~ 5	216 (58.7)	152 (41.3)	368 (45.3)	0.002	12.81 (2)
	6 ~ 10	141 (46.8)	160 (53.2)	301 (37.1)		
	≥ 11	64 (44.8)	79 (55.2)	143 (17.6)		
	**Total**	**421 (51.8)**	**391 (48.2)**	**812**		

^7^TOI, Turnover intention

### Association between safety culture, sexual harassment, working experience, sex, and turnover intention among nursing professionals

Logistic regression analysis was employed to examine the association of safety culture and workplace sexual harassment, adjusting for years of working experience and sex of nursing professionals, and further stratified by sex to examine whether the association differ. Table 4 presents results from the logistic regression models, model fit statistics, and variances. The Omnibus Test of the overall model coefficient (*N* = 812) confirmed the overall model (i.e., safety culture, workplace sexual harassment, years of working experience and sex) fit which is significantly better than the null model (χ² [6] = 48.746, *p* < 0.001), and the Hosmer and Lemeshow similarly indicated an adequate fit (χ² [8] = 7.239, *p* < 0.511). Safety culture, workplace sexual harassment, years of working experience and sex collectively accounted for approximately 7.8% (Nagelkerke R^2^ = 0.078) of the variance in turnover intention in the full sample. Given that sex of participants was a significant predictor in the overall model (Wald = 14.38, *p*<.001), sex-stratified logistic regression analyses were conducted to determine whether predictor patterns differ across sex.

The male (*n* = 412) Omnibus test of model coefficient was statistically significant (χ² [5] = 29.358, *p* < 0.001), indicating a model fit, and the Hosmer and Lemeshow test was not significant, indicating an excellent model fit (χ² [7] = 1.557, *p* < 0.980). Among females (*n* = 400), the Omnibus test of model coefficients was not statistically significant (χ² [5] = 10.114, *p* < 0.072), suggesting model misfit. The Hosmer and Lemeshow test was not significant (χ² [7] = 4.944, *p* < 0.667), indicating no evidence of model misfit, despite the predictors not significantly improving prediction beyond the null model for the female subgroup. Among males, the predictors explained 9.2% of the variance (Nagelkerke R^2^ = 0.092), driven primarily by safety culture (Wald = 17.347, *p* < 0.001). In contrast, among females, only 3.4% of the variance was explained, primarily by workplace sexual harassment (Wald = 5.576, *p* < 0.018).

As shown in Table 4, among all participants, nursing professionals working in facilities they perceived as having a pathological safety culture (aOR = 2.39, CI = 1.33–4.32) or bureaucratic safety culture (aOR = 1.80, CI = 1.30–2.48) compared with a generative safety culture were more likely to report high turnover intention. Being a male (aOR = 1.75, CI = 1.31–2.34) and having experienced high versus low sexual harassment were associated with a higher likelihood of reporting high turnover intention (aOR = 1.40, CI = 1.03 − 1.91). Meanwhile, ≤ 5 years of work experience was associated with a lower likelihood of reporting high turnover intention (aOR = 0.63, CI = 0.43 –0.94).

Sex-specific analyses revealed that sexual harassment was the only variable associated with turnover intention among females, indicating that high levels of sexual harassment were more associated with reported high turnover intention (aOR = 1.68, CI = 1.09–2.58). The overall female model did not reach statistical significance (*p* = 0.072); therefore, the associations observed for sexual harassment among females should be interpreted with caution. In contrast, sexual harassment was not associated with turnover intention among males. Instead, male nursing professionals working in facilities they perceived as having pathological (aOR = 3.60, CI = 1.50–8.65) or bureaucratic safety culture (aOR = 2.49, CI = 1.57–3.95) were more likely to report high turnover intention as compared with those working in generative safety culture facilities, while ≤ 5 years of work experience (aOR = 0.48, CI = 0.26 –0.88) was associated with a lower likelihood of reporting turnover intention.

**Table 4:** Binary logistic regression of safety culture, sexual harassment, working experience, and sex on turnover intention

	Variable		B	Wald	*p*-value	aOR^8^	95% CI^9^	
							Lower	Upper
	**Safety culture (SC)**							
	Pathological/Low SC		0.87	8.39	< 0.005	2.39	1.33	4.32
	Bureaucratic/Medium SC		0.59	12.81	< 0.001	1.80	1.30	2.48
	Generative/High SC		Ref^10^	Ref	Ref	Ref	Ref	Ref
	**Sexual harassment**							
	High		0.34	4.57	0.033	1.40	1.03	1.91
	Low		Ref	Ref	Ref	Ref	Ref	Ref
	**Years of Working Experience**							
	1–5 years		− 0.46	5.04	0.025	0.63	0.43	0.94
	6–10 years		− 0.16	0.59	0.444	0.85	0.56	1.29
	11 + years		Ref	Ref	Ref	Ref	Ref	Ref
	**Sex**							
	Male		0.56	14.38	< 0.001	1.75	1.31	2.34
	Female		Ref	Ref	Ref	Ref	Ref	Ref
Male	**Safety culture**							
	Pathological/Low SC		1.28	8.23	< 0.005	3.60	1.50	8.65
	Bureaucratic/Medium SC		0.91	15.03	< 0.001	2.49	1.57	3.95
	Generative/High SC		Ref	Ref	Ref	Ref	Ref	Ref
	**Sexual harassment**							
	High		0.16	0.47	0.49	1.17	0.75	1.83
	Low		Ref	Ref	Ref	Ref	Ref	Ref
	**Working experience**							
	1–5 years		− 0.73	5.66	0.017	0.48	0.26	0.88
	6–10 years		− 0.14	0.22	0.636	0.87	0.49	1.56
	11 + years		Ref	Ref	Ref	Ref	Ref	Ref
Female	**Safety culture**							
	Pathological/Low SC		0.49	1.34	0.247	1.63	0.71	3.70
	Bureaucratic/Medium SC		0.28	1.44	0.230	1.32	0.84	2.06
	Generative/High SC		Ref	Ref	Ref	Ref	Ref	Ref
	**Sexual harassment**							
	High		0.52	5.58	0.018	1.68	1.09	2.58
	Low		Ref	Ref	Ref	Ref	Ref	Ref
	**Years of Working Experience**							
	1–5 years		− 0.29	1.11	0.293	0.75	0.43	1.29
	6–10 years		− 0.31	1.02	0.313	0.73	0.40	1.34
	11 + years		Ref	Ref	Ref	Ref	Ref	Ref
	**Model summary**							
**Model**	**Cox and Snell (R** ^2^ **)**	**Nagelkerke** (R^2^)		**Omnibus χ²(df)**, *p***-value**			**Hosmer & Lemeshow** **χ²(df)**, *p*-**value**	
Overall (*N* = 812)	0.058	0.078		48.746(6), < 0.001)			7.239(8), = 0.511	
Males (*n* = 412)	0.069	0.092		29.358(5), < 0.001			1.557(7), = 0.980	
Female (*n* = 400)	0.025	0.034		10.114(5), = 0.072			4.944(7), = 0.667	

^8^aOR, adjusted odds ratio

^9^CI, Confidence interval

^10^Ref, reference category

## Discussion

This study examined the relationships among safety culture, workplace sexual harassment, and turnover intention among nursing professionals in Ghana’s Upper East Region. Findings indicate a markedly suboptimal safety culture across healthcare facilities in this predominantly rural area. Between 27% and 51% of nursing professionals reported high levels of sexual harassment—including gender harassment, unwanted sexual attention, and sexual coercion—while nearly half indicated elevated turnover intention. Although the full-sample model explained only 7.8% of variance in turnover intention (Nagelkerke R² = 0.078), the male model explained only 9.2% (Nagelkerke R² = 0.092), while the female model explained only 3.4% (Nagelkerke R² = 0.034). Thus, high sexual harassment and employment in facilities characterized by pathological or bureaucratic safety cultures were both associated with greater turnover intention. Among female nursing professionals, exposure to high sexual harassment was strongly linked to higher turnover intention, whereas among male nursing professionals, working within pathological or bureaucratic safety cultures was strongly associated with turnover intention. Considering that approximately two-thirds of the participants are married and the study was conducted in a more patriarchal context, adaptation, power dynamics, occupational role segregation, inadequate or lack of formal anti-sexual harassment enforcement, power asymmetry, and perception of hospital safety culture and sexual harassment may explain the sex-specific association observed.

### Level of safety culture

Strengthening safety culture is essential for retaining and attracting healthcare workers, particularly in low-resource settings. Our study found that 29.4% of participants reported a positive/generative safety culture, while the majority reported a bureaucratic safety culture. This finding suggests that whereas nursing professionals in the region perceive their facilities as adhering to safety and procedural standards, this adherence occurs without deeper engagement with workers. Thus, there is substantial room for improvement. Similar findings are found in other studies. For example, Italian nursing professionals working in primary hospitals funded by local health authorities reported overall safety culture at the bureaucratic level [48], and healthcare professionals, predominantly nursing professionals, in Brazil reported their workplace safety culture as below optimal levels [49]. These similarities highlight that bureaucratic safety cultures are common in low-resource settings.

Similar to our findings, another study of healthcare professionals in low-resource facilities in Indonesia indicated that 28.1% of respondents reported a generative safety culture [50]. In contrast, studies in well-resourced healthcare facilities in the United States of America and Saudi Arabia revealed better safety culture, with healthcare providers across 16 states in the United States of America rating their facilities as healthy and robust [51]. Moreover, an estimated 69% of nursing professionals in four hospitals in the Qassim Region of Saudi Arabia reported an excellent safety culture [52]. Variations in perceptions of safety culture may be associated with the instruments used to measure safety culture and demographics such as sex, level of education, and type of organization; less resourced facilities may have poorer safety culture.

### Prevalence of sexual harassment

Our study revealed alarming and unacceptable levels of sexual harassment among nursing professionals in the Upper East region of Ghana, with more than one-third of the respondents reporting a high level of sexual harassment at work. This finding significantly exceeds the global prevalence of sexual harassment among nursing professionals at 12.6% annually [24]. It also exceeds rates of sexual harassment reported in several systematic and meta-analyses globally, with prevalence ranging from 6% to 25% [53–58].

In Ghana, the prevalence of workplace sexual harassment among nursing professionals in the middle and southern zones has been reported in a range from 3.5% to 44%, disproportionately affecting lower-rank female nursing professionals; yet formal institutional policies addressing sexual harassment remain largely absent [59, 60]. Perpetrators include colleagues, supervisors, physicians, patients, and patients’ relatives [53, 60]. Our findings align with studies from Nigeria indicating that unwanted sexual attention is the most commonly reported form of sexual harassment among nursing professionals [61]. Although this pattern mirrors evidence from several African and other low- and middle-income country settings, research from many high-income countries identifies gender harassment as the predominant form of workplace sexual harassment [41, 42, 62–64].

Variation in sexual harassment prevalence across settings is influenced by demographic factors, contextual conditions, methodological differences, and organizational safety culture [21, 24, 55]. Regardless of prevalence, sexual harassment has wide-ranging consequences, including compromised patient care, increased medical errors, reduced quality of work, and significant mental, physical, emotional, and psychological burdens for nursing professionals. It also contributes to heightened turnover intention and imposes financial costs on healthcare facilities [21, 65].

Strengthened managerial commitment is therefore essential to prevent further deterioration and promote safer work environments. Implementing robust policies, clear reporting mechanisms, and comprehensive staff training on sexual harassment can help reduce turnover intention and enhance quality of care. Such actions are critical to advancing UHC and achieving SDG 3, ultimately fostering a safer and more equitable healthcare system for all.

### Level of turnover intention among nursing professionals

Safety culture, workplace sexual harassment, years of work experience, and sex collectively, modestly accounted for approximately 7.8% (Nagelkerke R^2^ = 0.078) of the variance in turnover intention in the full sample, 9.2% (Nagelkerke R^2^ = 0.092) among males, and 3.4% (Nagelkerke R^2^ = 0.034) among females. Despite the modest variance, it is consistent with the broader literature on turnover intention. For instance, turnover intention is associated with individual, interpersonal, organizational, and contextual factors [11, 15, 38, 66–68]. Using single-level or fewer associated factors often yields Nagelkerke R^2^ values ranging from 0.07 to 0.27 [69–72]. Our explained variances are therefore not atypical and within range and comparable to published studies among the healthcare workforce with sex differences in the experiences and consequences of sexual harassment and safety culture and climate perceptions. Possible explanations for the modest variance include omission of variables such as organizational commitment, perceived supervisor support, motivation, work-life balance, pay equity, burnout, leadership style, workplace bullying, psychosocial safety climate, and workplace violence [11, 15, 38, 66–68]. Their exclusion from our model, which focused theoretically on safety culture and workplace sexual harassment, likely contributed to the modest variance. Dichotomizing turnover intention into low and high categories likely reduced the variance captured relative to ordinal or continuous outcome models [69], despite being analytically appropriate for logistic regression and consistent with comparable studies. The substantially lower variance of 3.4% among females compared to 9.2% among males suggests that female turnover intention may be influenced by different patterns of factors outside of our model’s scope. For instance, domestic and caregiving responsibilities are significantly associated with female turnover intentions [73, 74]. In contrast, pay disparities by sex are associated with higher turnover rates among female healthcare professionals [75], although this is not the case in Ghana.

Nearly one in every two nursing professionals (48.2%) in healthcare facilities in the Upper East Region reported a high intention to leave, a figure similar to national and global trends. Previous studies in Ghana revealed similar estimates of turnover intention among nursing professionals in district and teaching hospitals [14, 25]. Similar findings are reported in other low- and middle-income countries with high turnover intentions (in some instances) and rates exceeding 70% [13, 15, 67].

Explanatory factors for high turnover intentions include a hostile work environment, poor staffing, limited career advancement, and salary disparities [76–78]. Contrary to our study’s findings, systematic reviews and meta-analyses have reported lower turnover intentions [9–12]. However, the higher turnover rates are largely from low- and middle-income countries such as Ghana, underscoring the challenges nursing professionals face in resource-limited settings.

Prior studies indicate that high worker turnover can negatively impact healthcare delivery and quality of patient care [16, 17]. Therefore, stakeholders in the Upper East Region’s healthcare sector should consider developing and implementing strategies that include enhancing workplace safety culture and implementing a zero-tolerance policy for workplace sexual harassment. These measures are critical to improving patient care and accelerating the achievement of UHC and SDG 3.

Similar to previous studies among nursing professionals, we found that males were more likely than females to have turnover intentions [58, 79–81]. Prior studies suggest that a societal perception of nursing as a “female” profession may be associated with male nursing professionals experiencing stigma, isolation, or role strain, thereby increasing their intent to leave their current positions [58, 82]. Nevertheless, we also found that different factors were associated with turnover intention in male and female nursing professionals.

Although males and females reported similar levels of pathological and bureaucratic safety culture, safety culture was associated with turnover intention only among male nursing professionals. Male nursing professionals’ perceptions of safety culture, coping, and adaptation to poor safety culture may account for the association observed only among male nursing professionals. For instance, males are less likely to report a positive perception of safety culture [83]. They may also consider it a reason to leave their current positions, compared to females who may have a higher risk of leaving their jobs due to interpersonal or psychosocial reasons such as sexual harassment. Additionally, given the patriarchal culture of the Upper East Region, where males have greater job mobility and empowerment and fewer family and caregiving responsibilities than females, male nursing professionals are more likely to have a higher turnover intention [84, 85]. Similarly, occupational role segregation, in which males are more likely to be assigned managerial roles, may contribute to differences in turnover intention compared to female colleagues [86].

Although a higher percentage of male nursing professionals reported sexual harassment, sexual harassment was associated with turnover intention among females only. We note, however, that the overall model for females did not reach statistical significance, and the association observed for sexual harassment among females should therefore be interpreted with caution. Considering that approximately two-thirds of the participants are married and the study is in a more patriarchal region, sex coping and adaptation to sexual harassment may possibly be linked to the observed association among only the female nursing professionals. Though there are no specific organizational role differentials for male and female nursing professionals in the region, we believe that the predominance of male superiors across hospitals, including physicians and physician assistants, demands that males pay particular attention to safety culture issues to protect females from sexual harassment. For instance, individual and family protection coupled with emotional and mental health outcomes, including humiliation, anger, symptoms of depression, anxiety, and impaired psychological well-being, may disproportionately affect female nursing professionals [87].

Additionally, fear of persistent coercion by perpetrators to secure their cooperation and the consequences of noncompliance may be associated with a higher intention for females than the males [88]. The absence of punitive anti-sexual harassment enforcement mechanisms in healthcare facilities [89] may amplify the impact of sexual harassment on female nursing professionals’ turnover intention. Female colleagues may also be more vulnerable relative to males, who tend to prioritize safety culture concerns [90]. Asymmetry of power where female nursing professionals are more likely to be lower cadre, such as community health nursing and auxiliary nursing roles, exposing them to sexual harassment from supervisors, colleagues, physician or patients and their relatives [91]; coping strategies and mechanisms, where females may possibly internalized sexual harassment more acutely as an interpersonal threat that impacts their professional identity and psychological safety [92]. The observed association may also reflect a higher percentage of a specific subscale of sexual harassment analyzed in this study. Whereas prior studies indicate that sexual harassment is associated with higher turnover intention [93, 94], these studies did not stratify by sex.

Considering that a high level of sexual harassment is associated with higher turnover intention among nursing professionals, the Ghana Health Service and all directors of health in the region must commit to interventions that prevent and have zero tolerance for sexual harassment. The strategies should include creating confidential reporting systems to encourage victims to report sexual harassment incidents. Other studies have reported negative impacts of sexual harassment on nursing professionals’ psychological health [94], sleep quality, depression, overall health, and well-being [95], which may in turn lead to higher turnover intention. Thus, there is an urgency to implement measures to prevent workplace sexual harassment, especially among female nursing professionals.

It is possible that feelings of stigma, isolation, or role strain have contributed to the high prevalence of a pathological safety culture rating among male nursing professionals in our study, which may possibly be associated with the relationship between safety culture and turnover intention among males. Among males in facilities with pathological and bureaucratic safety cultures, compared with generative safety cultures, their likelihood of turnover intention was approximately 3.6 times and 2.5 times, respectively.

Additionally, nursing professionals with a few years of work experience were less likely to have turnover intentions, with a similar pattern among male nursing professionals. This pattern is contrary to reports from several systematic reviews and meta-analyses [9, 12, 24], but aligns with patterns observed in other primary studies [96, 97]. Importantly, the existing literature has primarily treated sex as a covariate rather than conducting sex-sensitive analyses, underscoring the originality and contextual relevance of our study’s approach. Nurses with fewer years of working experience tend to have higher job engagement and motivation [98], undergo organizational socialization [99], and receive support and training to adapt to their facilities [100]. For instance, very recent evidence showed that Kenyan nursing professionals aged 21–40 and those with less than 5 years of practice were significantly less likely to leave their current jobs than those aged 41–60 [101]. These workers also benefit from professional development opportunities, such as mentorship, continuing education, and skill-building, which may protect them from turnover intentions [102]. Similar findings are reported in other studies conducted in low-to-middle-income Sub-Saharan countries [13, 97]. Evidence from systematic reviews and meta-analyses further showed that turnover and turnover intention are high in Africa; providing financial and non-financial incentives, increased training of nurses, steering students to shortage specialties, adequate rural housing, facility-level improvements, professional progression opportunities, nurses’ recognition and involvement, and supportive work environments could minimize it [66, 103]. Considering that nursing professionals in Ghana self-select their regions of posting, those with fewer years of work experience may be enjoying their self-selected regions and facilities, making them less likely to have turnover intentions.

Our findings highlight an interrelationship among organizational factors such as safety culture, interpersonal dynamics, including experience of sexual harassment, and personal factors such as sex and years of experience, which influence turnover intention. This highlights the need for urgent and specific intervention from the personal to organizational levels. Zero-tolerance policies for sexual harassment in healthcare facilities with a robust safety culture transition from bureaucratic to generative safety culture practices are needed. Moreover, instituting a specific confidential reporting system, such as an online platform, could be an essential strategy to strengthen safety culture, reduce sexual harassment, attract and retain nursing professionals to improve healthcare quality, and achieve UHC and SDG 3. Future studies are needed to determine whether addressing the weak safety culture in the Upper East Region of Ghana could support healthcare providers in promoting high-quality patient care and in influencing the achievement of UHC and SDG 3.

### Strengths and limitations

The study’s strengths include sex-stratified analyses, while the online survey facilitated broader access to potential participants. Moreover, the study provided a comprehensive assessment of safety culture, workplace sexual harassment, and turnover intention of nursing professionals from rural healthcare settings. Despite the study’s strengths, we acknowledge the following limitations. Firstly, the cross-sectional design of the study precludes causal inference regarding the relationships among sexual harassment, safety culture, and turnover intention. The study included only public healthcare facilities where nursing professionals are on government payroll (i.e., GHS and CHAG facilities), excluding private facilities in the region. Therefore, the findings may not be generalized beyond GHS and CHAG healthcare facilities in the Upper East Region. Similarly, the regional patriarchal norms may limit the transferability to other Ghanaian or African contexts.

Although we attempted to reach all the nursing staff in the region, only 22.2% responded. While the 22% response rate (812 participants) may provide a sufficiently powered analytic sample, it precludes a strong claim of representativeness and generalizability to the target population. Additionally, reliance on online surveys, particularly on WhatsApp platforms, may have introduced digital access bias, as nursing professionals from sub-remote districts with poor internet connectivity, those without smartphones, those not using WhatsApp, those unable to afford internet access, or those with lower digital literacy may have been systematically excluded. This could possibly skew the sample towards younger, more urban, and digitally literate nursing professionals. Therefore, future studies should complement online surveys with paper-based or interview-administered alternatives in rural Ghanaian settings.

The frequency of sexual harassment over a period of a year is susceptible to recall bias, as participants may not be able to remember all incidents of sexual harassment if they had frequent experiences. Similarly, considering the sensitive nature of sexual harassment, particularly in a patriarchal and predominantly rural context where reporting may be suppressed, social desirability bias or underreporting bias are likely. Our model dichotomized turnover intention into low/high, which may not have captured nuances beyond those of ordinal or continuous models. We also acknowledge that there may be multiple entries, as we only cautioned participants not to complete the survey more than once. We recommend measures such as IP address tracking, sending unique survey links to individual participants, or implementing login-based access controls in subsequent online surveys to minimize duplicate entries. Despite the theoretical and practical significance of safety culture, workplace sexual harassment, years of working experience and sex collectively accounting for turnover intention, their variance is modest-approximately 7.8% of turnover intention in the full sample, 9.2% among males, and 3.4% among females. The female model did not achieve global significance; therefore, the results should be interpreted with caution. Future research should integrate a broad range of comprehensive antecedents of turnover intention. Future studies of the entire country of Ghana, including government payroll healthcare facilities and private healthcare facilities, should be conducted.

## Conclusion

The study highlights a suboptimal safety culture characterized by a high-level bureaucratic culture in healthcare facilities in Ghana’s Upper East Region. Unfortunately, these workers reported an unacceptably high level of sexual harassment and a high level of turnover intention. It is not surprising that large numbers of nursing professionals are leaving Ghana’s healthcare sector. Therefore, prioritizing the development and implementation of targeted strategies, including policies to improve safety culture and address sexual harassment, while accounting for years of experience and sex differences, is critical. Despite the overall model for female nursing professionals not reaching significance, the associations between sexual harassment and turnover intention deserve further examination and targeted policy attention. Therefore, prioritizing prevention and reporting of sexual harassment may be critical to protect female nurses and reduce their turnover intention. Among males, prioritizing interventions and strategies should focus on improving hospital safety culture and the work experience to reduce turnover intention. Managers of GHS and CHAG healthcare facilities in Ghana’s Upper East Region should therefore develop and implement strategies and interventions to foster a positive safety culture to reduce sexual harassment, particularly unwanted harassment, to mitigate turnover intention among nurses in the region. Specifically, anonymous digital reporting systems, such as short message service (SMS) or unstructured supplementary service data (USSD), should be implemented and accessible in low-connectivity settings for reporting workplace sexual harassment incidents, and should also protect the victim. Routine safety culture audits using standardized instruments, with facility benchmarks based on facility type, and feedback to facilities and their managers to aid transition from bureaucratic to generative safety practices could be effective. Similarly, purposive involvement of staff in safety committees at ward or department levels in open error reporting norms and biannual safety culture reviews, as part of accreditation mandates under the GHS and CHAG directorate, is essential. Mandatory sex-sensitive workplace harassment training for all staff and supervisors, integrated into the existing continuing professional development framework and accompanied by clear punitive measures, is recommended to mitigate sexual harassment incidents.

## Data Availability

Data are not publicly available, however on reasonable request from the corresponding author they can be provided with permission from Institutional Review Board of the University of Cape Coast and Navrongo Health Research Center.
